# Effect of Interlayer Temperature on Microstructure and Properties of High-Strength Low-Alloy Steel Manufactured Using Submerged-Arc Additive Manufacturing (SAAM)

**DOI:** 10.3390/ma17215376

**Published:** 2024-11-03

**Authors:** Meijuan Hu, Qiang Chi, Lingkang Ji, Weiwei Li, Shuai Yan, Fangjie Cheng

**Affiliations:** 1 State Key Laboratory of Oil and Gas Equipment, CNPC Tubular Goods Research Institute, Xi’an 710077, China; chiq@cnpc.com.cn (Q.C.); jilk@cnpc.com.cn (L.J.); liweiwei001@cnpc.com.cn (W.L.); 2 School of Materials Science and Engineering, Tianjin University, Tianjin 300350, China; ys0423@tju.edu.cn (S.Y.); chfj@tju.edu.cn (F.C.); 3 Tianjin Key Laboratory of Advanced Joining Technology, Tianjin 300350, China

**Keywords:** SAAM, interlayer temperature, microstructure, mechanical properties

## Abstract

Controlled interlayer temperature has a profound impact on both the microstructure and mechanical properties of the deposited components. In this study, thin-walled structures made of high-strength low-alloy steel were fabricated using the submerged-arc additive manufacturing process. The effects of varying temperature on the microstructure and mechanical properties of the components were studied. The results showed that the cooling rate within T_8/5_ decreased as the interlayer temperature increased, which caused the microstructure to transition from a fine-grained structure dominated by bainitic ferrite and granular bainite to a coarse-grained structure dominated by polygonal ferrite. The measurement of mechanical properties showed that due to the influence of the fine-grained structure, the components with low interlayer temperatures exhibit excellent hardness, high strength, and outstanding ductility and toughness. Furthermore, a faster cooling rate disrupts the stability of carbon diffusion, resulting in the development of increased quantities of residual austenitic films within the components with controlled low interlayer temperatures. This augmentation in residual austenite films strengthens the components’ ductility and toughness, enabling the deposited components to exhibit exceptional impact toughness in low-temperature environments.

## 1. Introduction

The homogeneity of microstructures, isotropy of mechanical properties, and coordination of large-scale structures are essential for manufacturing large-scale oil and gas equipment made of low-alloy steel. Liu et al. [1] developed a disruptive steel with an ultrahigh yield strength (YS) close to 2 gigapascals (GPa) and outstanding fracture toughness by activating delamination toughening coupled with transformation-induced plasticity. Sun et al. [2] broke the trade-off between strength and toughness by annealing low-alloy bainitic steel in the two-phase region of hot rolling to form an ultrafine-grained ferrite/martensite lamellar microstructure. In addition, thermally treated components not only exhibit anisotropic mechanical properties but also display heterogeneous microstructures along the thickness direction [3]. Hong et al. [4] studied the effect of microstructural changes on the Charpy impact properties of thick-walled Mn-Mo-Ni low-alloy steel using a 210 mm thick reactor pressure vessel. The results revealed that the ductile-to-brittle transition temperature varied across the component, with −5.6 °C at the surface, 11.5 °C in the lower region, and 6.8 °C in the middle. The formation of this heterogeneity was attributed to temperature or stress variations within the component during heat treatment, rolling, or forging, leading to inhomogeneous microstructures and mechanical properties from the surface to the center [5]. However, few studies have matched and integrated these three aspects.

Additive manufacturing (AM) technology has revolutionized production based on the “discrete accumulation” principle. It creates intricate three-dimensional parts by depositing the material in two-dimensional planes, achieving a near-net shape forming of parts [6]. Wire-arc additive manufacturing (WAAM) has greater flexibility, higher deposition efficiency, better economic benefits, and reduced environmental effects compared to casting [7,8,9,10,11,12], selective laser melting [13], direct energy deposition [14], and aminated object manufacturing [15]. However, in processes such as open-arc WAAM (including gas metal arc welding, gas tungsten arc welding, and plasma arc welding), the molten metal solidifies naturally during the manufacturing process. This natural solidification can give rise to defects like dripping, slag entrapment, porosity, and incomplete fusion [7,16,17]. Playing the open-arc WAAM process on materials with strong epitaxial growth tendencies frequently results in parts that display inhomogeneity and anisotropy in their mechanical properties [18,19,20].

Submerged-arc additive manufacturing (SAAM) is derived from submerged-arc welding. The slag acts as a restraint on the freely flowing molten metal during the solidification of the molten granular flux in the manufacturing process [21]. Furthermore, it is characterized by inherent “full-layer penetration” intrinsic heat treatment (IHT) and boasts high deposition efficiency. Li et al. [22]. utilized the inherent thermal effects of SAAM to produce low-carbon steel deposit walls with fully equiaxed ferrite. These deposit walls exhibited excellent isotropic properties in Charpy impact tests conducted at −60 °C. Hu et al. [23] prepared a high-strength low-alloy (HSLA) steel T-branch pipe using SAAM and found that it exhibited excellent isotropic properties in the tensile test. However, heat dissipation is primarily conducted through the substrate in the SAAM process. This leads to severe heat accumulation due to a heat input of several kJ/mm, significantly negatively impacting the microstructure and mechanical properties of the deposited samples [24,25]. Therefore, the optimization of process parameters and deposition strategies has become an urgent challenge to be addressed [26,27,28].

Interlayer temperature is a crucial factor affecting the microstructure of manufactured parts [29,30,31]. In this study, HSLA steel thin-walled components were successfully prepared with precise control of interlayer temperatures at 100 °C and 200 °C by SAAM. The influence of interlayer temperature on the microstructure and mechanical properties of HSLA steels was investigated, revealing the underlying mechanisms through which interlayer temperature affects the mechanical properties of the components. This provides significant theoretical insights for optimizing process parameters in SAAM.

## 2. Materials and Methods

### 2.1. Materials and SAAM Procedures

In the manufacturing process of SAAM components, the equipment included a custom-designed welding torch installed on a three-axis positioning system and an Aotai MZ 1000-IV SAW(Aotai, Jinan, Shandong, China) guided by a three-dimensional programmable control module. Before the experiments commenced, the substrate was meticulously ground to remove surface contaminants and oxide films and then cleaned thoroughly with anhydrous ethanol. The substrate was then securely clamped in a specially designed fixture before welding. The GWR-WEF1 with a diameter of 4.0 mm and GXL-125 fluxes were used as the local embedding medium and deposition material, respectively. Table 1 shows the nominal chemical composition of the welding wire.

Prior to arc ignition, the particle-based flux was pre-deposited and collected in a funnel. To maintain the integrity of each layer, an alternating scanning strategy was employed. After each layer was deposited, the slag generated during solidification was manually removed. Then, the welding torch was adjusted to the same horizontal height as the current deposition layer. Throughout this process, an infrared pyrometer (Keyence-FT-H50K, KEYENCE, Shanghai, China) was used to measure the temperature, and the accuracy of this pyrometer was ±3 °C. The distance between the infrared pyrometer and the component was 5 cm. The deposition step was repeated when the interlayer temperature reached the desired experimental conditions of 100 °C or 200 °C. K-type thermocouples were sequentially spot-welded manually on the side of the 20th layer of the single-wall deposition. The accuracy of K-type thermocouples was an ±0.75% T (where T is the measured temperature), and the frequency was 2 Hz. In addition, the thermocouple may fall off during the AM process. Therefore, a 4-channel thermocouple was used at equally spaced positions at the same horizontal height for measurement. The process parameters used in the experiments are detailed in Table 2. These parameters were determined through multiple preliminary experiments and encompass key factors such as welding current, welding voltage, scanning speed, wire feed rate, and wire extension length.

### 2.2. Microstructure Characterization

**Figure 1:**
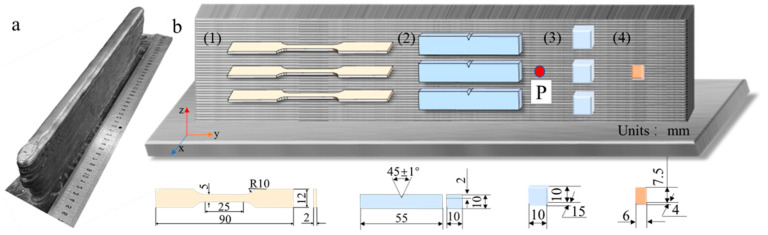
(**a**) Finished deposit parts; (**b**) extraction location of tested specimens: (**1**) tensile samples; (**2**) Charpy impact test samples; (**3**) metallographic samples; (**4**) electron backscatter diffraction (EBSD) sample; P: thermal cycle test location.

The metallographic samples of Figure 1(b3) were ground and polished to remove surface impurities and marks and then etched with a 4% solution of nitric acid–alcohol. Scanning Electron Microscopy (SEM, using a JEOL-7800F, JEFO, Tokyo, Japan) was utilized to observe the microstructures. To reveal the crystallographic information of the deposited parts, an EBSD sample was extracted, as shown in Figure 1(b4). Under the conditions of 30 V voltage and 23 °C, the EBSD sample was electropolished using a mixed solution of 5% perchloric acid and 95% anhydrous ethanol.

### 2.3. Mechanical Testing

To test the mechanical properties, samples were taken from the deposited parts. The tensile samples were machined into dog-bone shapes in accordance with GB/T 228-2002, as shown in Figure 1b. A 100 kN electronic universal testing machine (MST E45.105, Beijing, China) was used to test the tensile properties of the SAAM samples at room temperature, with a strain rate of 2.0 × 10^−3^ m/s achieved using a 25 mm extensometer(MST, Beijing, China). According to the GB/T 229-2020, standard full-size impact samples (10 × 10 × 55 mm) were selected, with the notch positioned at the interlayer fusion line. Prior to impact testing, the samples were held at −40 °C for 15 min and then tested using a pendulum impact tester (ZBC2752-ED, MST, Beijing, China). The impact fractures of the sample were observed by SEM. Using a DHV-1000 micro-Vickers hardness tester (UnitedTest, Beijing, China) under the conditions of a load of 100 N and a loading time of 15 s, the hardness of the sample was measured in the Y-Z plane and the X-Y plane, with a distance of 500 μm between indentations.

## 3. Results and Discussion

### 3.1. Thermal Analysis

**Figure 2:**
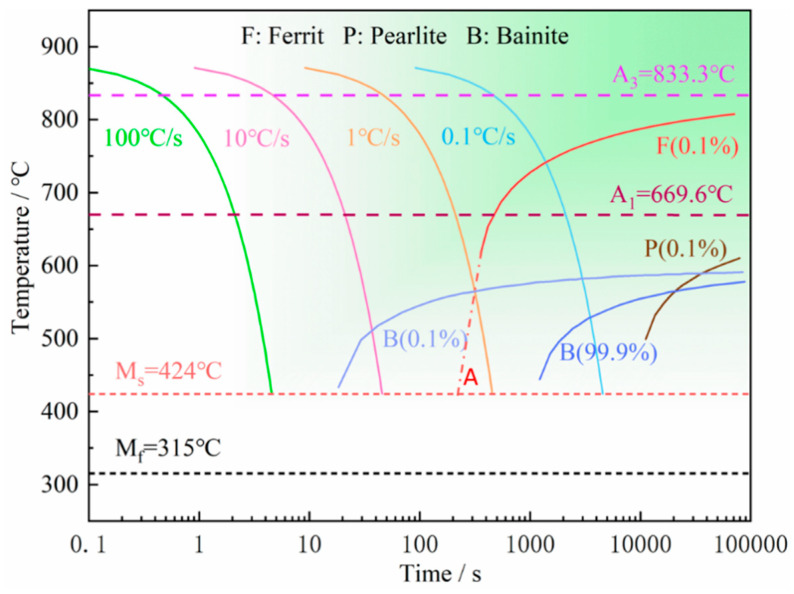
CCT calculated based on the chemical composition of the GWR-WEF1 wire.

The continuous cooling transformation curve (CCT) for the thermodynamic calculation for cooling process analysis was simulated by JMatPro software (Version7.0), based on the chemical composition of the GWR-WEF1 wire. The austenitizing temperature (A_c3_) was 833.3 °C, as shown in Figure 2. Austenite is transformed into various morphologies of ferrite within the temperature range of 800–500 °C. Polygonal ferrite is formed at a cooling rate below 1 °C/s. Bainitic ferrite is formed at a cooling rate ranging from 1 to 10 °C/s within the temperature range of 550–450 °C, and austenite transforms directly into bainitic ferrite at higher cooling rates within this range. In addition, polygonal ferrite continues to be formed within the bainite transformation range. Additionally, martensite forms at a cooling rate that exceeds 10 °C/s.

**Figure 3:**
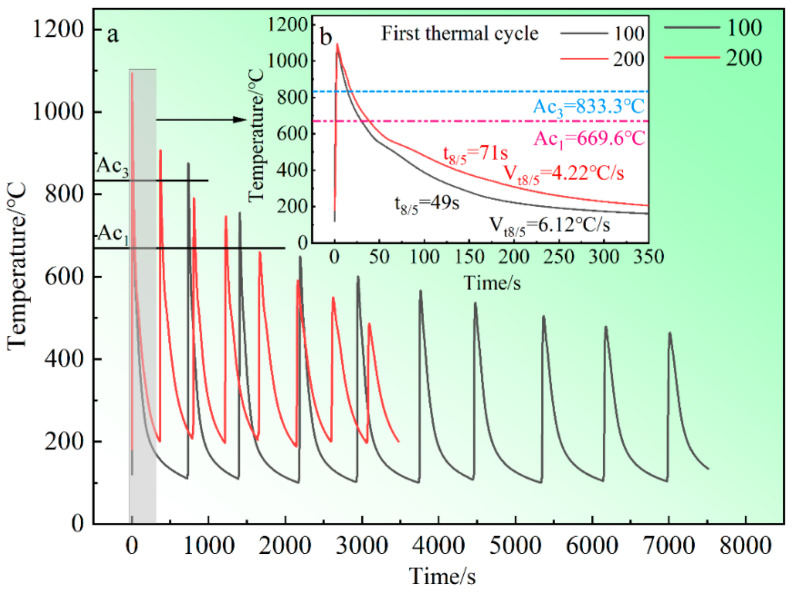
The thermal cycles of SAAM. (**a**) The thermal cycle detection curve of components at different interlayer temperatures, (**b**) The cooling rate of the component at the first thermal cycle.

The components fabricated by SAAM underwent continuous solidification, complete austenitizing, partial austenitizing, high-temperature tempering, and low-temperature tempering due to the multiple thermal cycles (Figure 3a). In addition, higher interlayer temperatures have more heat accumulation within the deposited layers and enhance the thermal effect of subsequent deposited layers on the completed ones, at the same heat input. The peak temperatures are the same at different interlayer temperatures at the first layer, as shown in Figure 3a. But during subsequent thermal cycles, the peak temperatures at an interlayer temperature of 200 °C are always higher than those at 100 °C within the range of 800–500 °C. Moreover, the peak temperature is below A_c1_ at an interlayer temperature of 100 °C, while it remains within the range of A_c1_ to A_c3_ at 200 °C at the deposition of the fourth layer.

Additionally, high heat accumulation results in a slower cooling rate compared to low heat accumulation. The cooling rate is 4.22 °C/s at an interlayer temperature of 200 °C, whereas it reaches 6.12 °C/s at 100 °C, as shown in Figure 3b. For HSLA steels, the austenite grains grow significantly within the temperature range of 1300–1100 °C. Within the range of 800–500 °C, austenite transforms into different morphologies of ferrite. Furthermore, the cooling rate within the range of 800–500 °C determines the microstructure type of HSLA steels [32]. Hence, controlling the interlayer temperature can effectively regulate the microstructure of the components.

### 3.2. Microscopic Characterization

#### 3.2.1. Microstructure

**Figure 4:**
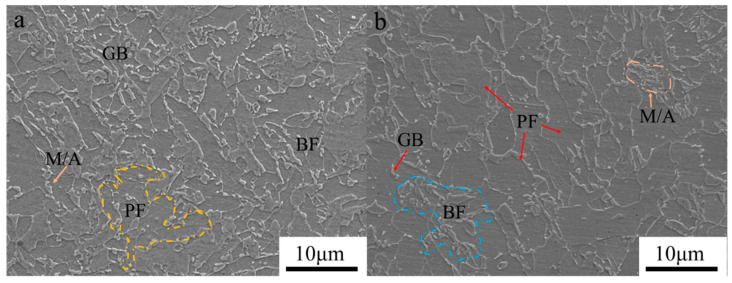
Microstructure: (**a**) 100 °C interlayer temperature and (**b**) 200 °C interlayer temperature.

The microstructures in the middle region of the components fabricated under different interlayer temperatures are depicted in Figure 4. Crystal morphologies with different interlayer temperatures exhibit a typical massive structure. Also, the granular grains have disappeared and polymorphic grains have formed with the increase in interlayer temperature. According to the morphology, the massive microstructure can be identified as bainitic ferrite (BF), the granular grain can be identified as granular bainite (GB), and the polygonal grain can be identified as polygonal ferrite (PF). Thus, the microstructure at 100 °C is primarily composed of BF and GB, as shown in Figure 4a. At an interlayer temperature of 200 °C, the microstructure mainly comprises PF and BF, as shown in Figure 4b. Song et al.’s [25] research also indicated that a decrease in cooling rate leads to the formation of quasi-polygonal ferrite with the same heat input conditions. Low interlayer temperatures provide lower heat accumulation, which results in a relatively fast cooling rate for the components. This inhibits diffusion-controlled PF transformation and favors the non-diffusion bainite transformation, thereby promoting the formation of a bainitic structure [33]. In addition, the supercooling and driving force for the austenite-to-bainite transformation increase at faster cooling rates, resulting in the formation of a finer bainite structure [34]. Conversely, heat accumulation in the deposited layer increases at high interlayer temperatures, resulting in a decrease in cooling rate. This inhibits the nucleation of bainitic grains and promotes the diffusion-driven growth of PF grains [35].

#### 3.2.2. Crystallographic

**Figure 5:**
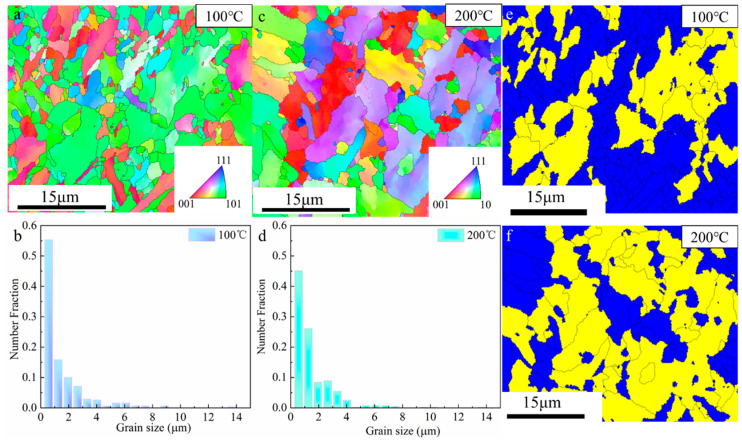
(**a**,**c**) Inverse pole figure-Z map; (**b**,**d**) grain size distribution; (**e**,**f**) grain orientation spread map (yellow: recovery, blue: recrystallization).

**Figure 6:**
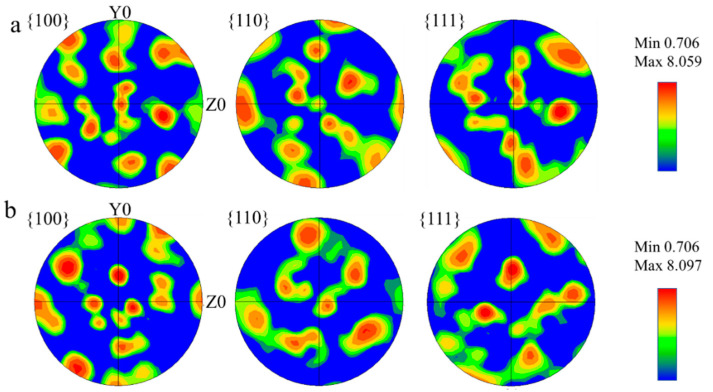
Pole figure maps of SAAM-deposited materials. ((**a**): interlayer temperature of 100 °C, (**b**): interlayer temperature of 200 °C.)

EBSD inverse pole figures and grain orientation spread maps of the components under different interlayer temperatures are shown in Figure 5. It can be seen in Figure 5a,c that the grain along the *Z*-axis direction is without significant orientation. This is attributed to the IHT mechanism of SAAM. Xu et al. [36] also showed that the double allotropic transformation (from δ-ferrite to austenite and from austenite to α-ferrite) experienced by lower-alloy steels tends to induce an almost non-oriented microstructure. Furthermore, the maximum textures at interlayer temperatures of 100 °C and 200 °C were observed to be 8.059 and 8.097, respectively (Figure 6). These data are attributed to the solid-state phase transformation process, wherein a specific Kurdjumov–Sachs (K-S) and Nishiyama–Wasserman (N-W) orientation relationship has been proven to exist between austenite and bainite [37,38].

The number of grain sizes within the 0–1 μm range accounted for 55% at an interlayer temperature of 100 °C, whereas at 200 °C, it only accounted for 45% (Figure 5b,d). Shalini et al. [39], utilizing CMT heat sources for WAAM, also demonstrated that walls constructed at lower interlayer temperatures exhibit finer grain structures compared to samples constructed at higher interlayer temperatures. In addition, small-sized grains have significant deformation resistance and high deformation storage capacity, which is conducive to the occurrence of recrystallization. As shown in Figure 5e,f, 61% of the microstructure underwent recrystallization at an interlayer temperature of 100℃, whereas at an interlayer temperature of 200 °C, only 37% underwent recrystallization.

**Figure 7:**
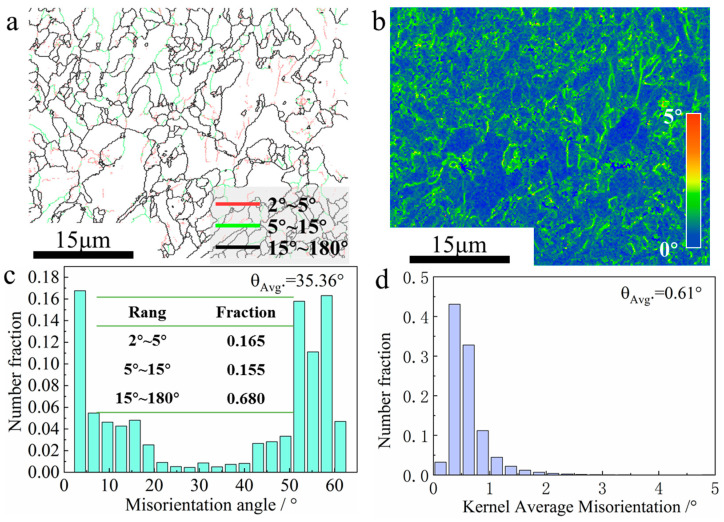
Microstructural evolution of deposits at 100 °C interlayer temperature: (**a**) grain boundary; (**b**) KAM; (**c**) statistical diagram of orientation difference angle; (**d**) KAM statistical diagram.

**Figure 8:**
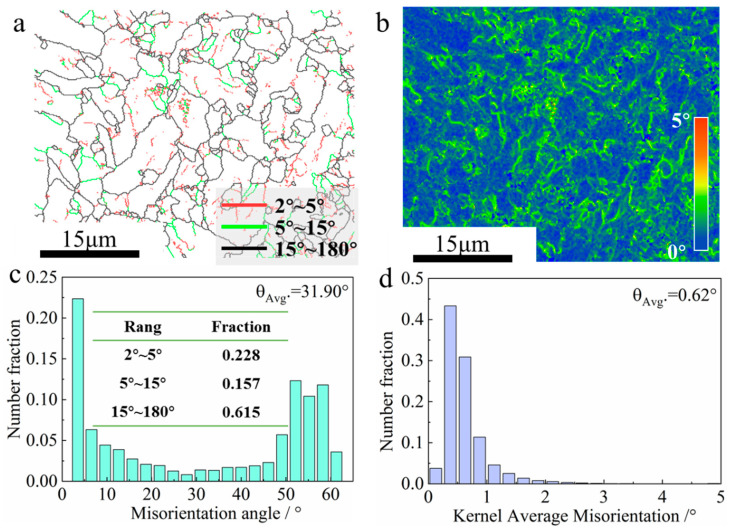
Microstructural evolution of deposits at 200 °C interlayer temperature: (**a**) grain boundary; (**b**) KAM; (**c**) orientation difference angle statistical diagram; (**d**) KAM statistical diagram.

The content of low-angle grain boundaries (LAGBs) increased from 32% to 38.5% when the interlayer temperature was controlled and rose from 100 °C to 200 °C, as shown in Figure 7a and Figure 8a. The high heat accumulation at an interlayer temperature of 200 °C promoted the formation of LAGBs with misorientation angles below 15° [40]. The average misorientation angle *θ_Avg_* decreased from 35.36° to 31.90°, as shown in Figure 7c and Figure 8c. The lower cooling rate enhanced the driving force for solid-phase transformation, leading to the gradual enlargement of austenite grains and a tendency to transform into a ferrite microstructure rather than a bainitic microstructure [41]. Consequently, fewer high-angle grain boundaries (HAGBs) were formed [34].

Kernel average misorientation (KAM) represents the average orientation difference between a given point and all its adjacent points. It is usually used as a measure of the local strain (strain storage energy) level and is an important indicator of the density of geometrically necessary dislocations (GNDs) [42]. Equations (1) and (2) give the relationship between GND density and KAM value and local strain, respectively.

(1) $$\rho_{GNDs}=\frac{\alpha \theta }{bd_{0}}$$

(2) $$E_{s}\approx \frac{1}{2}G\rho_{GNDs}b^{2}$$

where $\rho_{GNDs}$ represents GND density, *θ* (rad) denotes the average KAM value across the dislocation boundary, *b* signifies the magnitude of the Burgers vector (with *b* = 0.283 nm), *d*_0_ is the average spacing between all dislocation boundaries, and *α* is a constant (for pure tilt and twist GBs, *α* could be taken as 2 and 4, respectively). *E_s_* represents the strain energy stored per unit volume, and *G* stands for the shear modulus.

Figure 7d and Figure 8d indicate that the *θ_Avg_* of KAM at interlayer temperatures of 100 °C and 200 °C is 0.61 and 0.62, respectively. The value of $\rho_{GNDs}$ is approximated based on varying interlayer temperatures, according to Equation (1). $\rho_{GNDs}$ is considered an indication of the degree of incompatibility between phases. It is generally accepted that GNDs are caused by the filling or excavation of voids and overlapping regions in the lattice arrangement, resulting from dislocation motions between phases [43]. The value of $E_{s}$ is also approximated based on varying interlayer temperatures based on Equations (1) and (2). In summary, the degree of distortion within the components prepared at different interlayer temperatures is similar.

### 3.3. Mechanical Properties

#### 3.3.1. Microhardness

**Figure 9:**
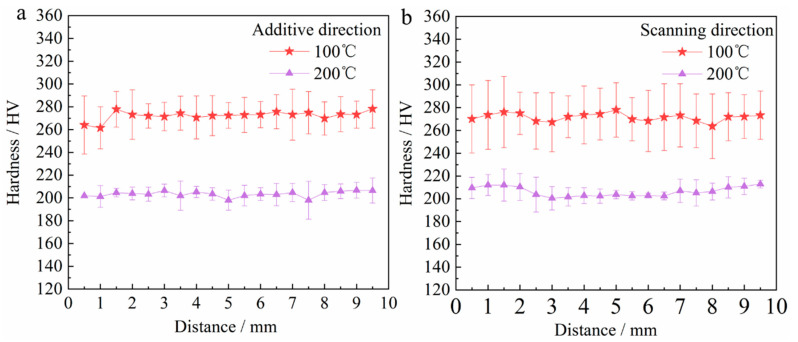
Microhardness distribution diagram. (**a**) Microhardness along the additive direction. (**b**) Microhardness along the scanning direction.

The microhardness distribution of the components under different interlayer temperatures are shown in Figure 9. In Figure 9a, the microhardness distribution along the Z axis ranges from 240 to 300 HV10, at an interlayer temperature of 100 °C. At 200 °C, it ranges from 180 to 210 HV10. The microhardness distribution along the Y axis varies between 240 and 310 HV10 at an interlayer temperature of 100 °C and between 190 and 220 HV10 at 200 °C, as shown in Figure 9b. Through comparison, it is observed that the microhardness at an interlayer temperature of 100 °C is higher than that at 200 °C. As shown in Figure 3 and Figure 4, the inherently rapid cooling rate associated with the lower interlayer temperature results in the formation of a larger proportion of a fine bainite microstructure, and the amount of bainite is positively correlated with the microhardness of the material [44]. In addition, the lower cooling rate at an interlayer temperature of 200 °C facilitates grain growth, leading to an enlargement of the total grain boundary area and a reduction in their number. The enlargement of grain spacing results in reduced mutual interference and a weaker ability to coordinate deformation, ultimately leading to a decrease in the hardness value of the deposited component. Furthermore, there is minimal variation in microhardness along both the Z and Y axes, suggesting a uniform and relatively isotropic microstructure throughout the deposited layer.

#### 3.3.2. Uniaxial Tensile Tests

Table 3 reveals that the component exhibits a yield strength (YS) of 560 MPa at an interlayer temperature of 100 °C, which exceeds the 504 MPa observed at 200 °C by approximately 56 MPa. However, Rafieazad, M et al. [45] fabricated a single-walled wall made of ER70S-6 HSLA steels using GMAW, with a yield strength of 400 MPa. Furthermore, the elongation (EL) of the component deposited at 100 °C stands at 32.8%, significantly surpassing the 21.6% recorded at 200 °C by roughly 10%. These data strongly indicate that as the interlayer temperature increases, the strength and plasticity of the deposited parts decrease. A high proportion of fine-grained and recrystallized structures was formed at an interlayer temperature of 100 °C, as shown in Figure 5e,f. A fine-grained structure not only enhances the strength of grain boundaries but also improves the ductility of the component [46]. Normally, grain size plays a significant role in the mechanical properties of metallic materials, which can be summarized by the Hall–Petch relationship [47,48]:

(3) $$\sigma_{s}=\sigma_{0}+K_{y}d^{-1/2}$$

where $\sigma_{s}$ is yield strength, $\sigma_{0}$ is friction stress, $K_{y}$ is a constant, and d is grain size. According to Equation (3), the smaller the grain size, the better the mechanical properties.

In addition, the ultimate tensile strength (UTS) of the deposited component at an interlayer temperature of 100 °C is approximately 18 MPa higher than that at an interlayer temperature of 200 °C. Studies have shown that there is a typical linear correlation between microhardness and UTS [49].

#### 3.3.3. Charpy Impact Tests

**Figure 10:**
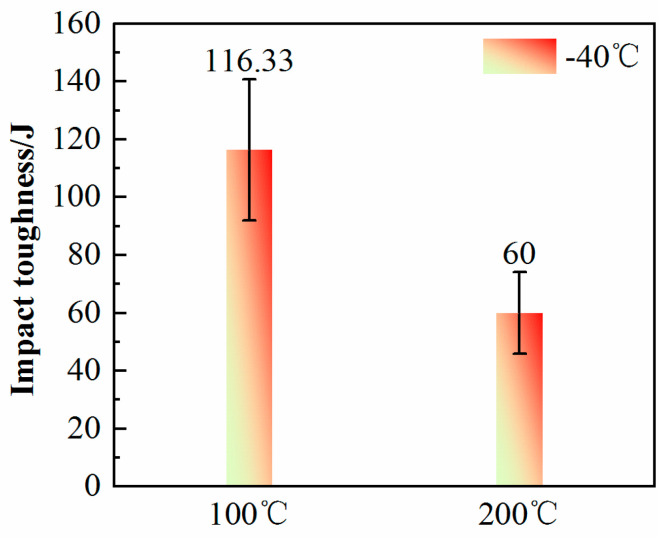
The impact-absorbed energy obtained by instrumented impact test at −40 °C (three specimens were used for each group in the impact test).

**Figure 11:**
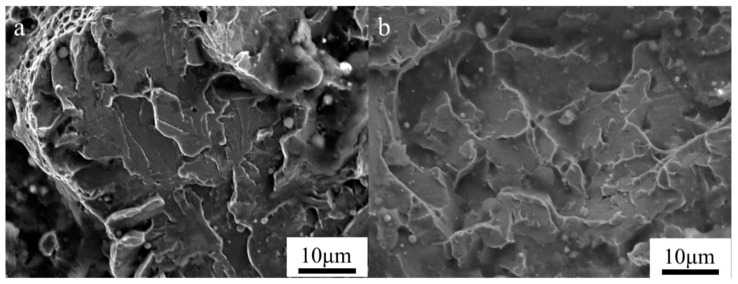
SEM of the fractured surface of specimens tested along the longitudinal direction ((**a**): interlayer temperature of 100 °C; (**b**): interlayer temperature of 200 °C).

The impact absorption energy of the deposition parts produced at an interlayer temperature of 100 °C with a low-temperature condition of −40 °C is 116.33 ± 20 J, whereas that of the deposition parts at 200 °C is 60 ± 15 J, as shown in Figure 10. This significant difference further confirms that deposition parts obtained at a lower interlayer temperature exhibit superior toughness. However, Figure 11 also indicates that there is a scatter in the toughness data of the components, and the dispersion of toughness data at 100 °C is greater than that at 200 °C. This is attributed to the fact that the as-deposited layer is to be reheated by the subsequent deposited layer to a temperature range defined by the a + γ phase field region. Austenite is formed at PAGBs and bainitic lath boundaries [50,51], and it further grows through carbon enrichment and transform into martensite–austenite (M/A) constituents upon faster cooling or into polygonal ferrite upon slower cooling [17,52]. Figure 4 clearly shows the presence of M/A constituents at the grain boundaries.

**Figure 12:**
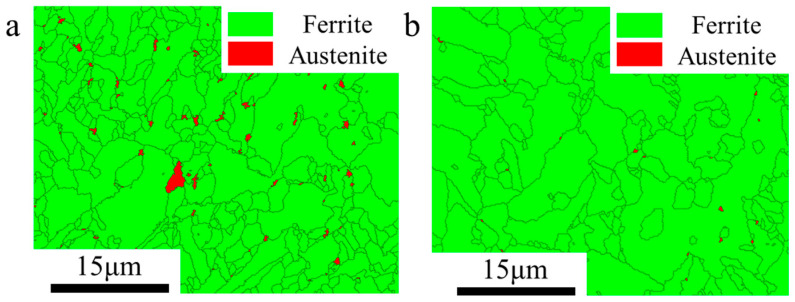
Phase map ((**a**): interlayer temperature of 100 °C; (**b**): interlayer temperature of 200 °C).

The fractured surface of the samples of the components under different interlayer temperatures are shown in Figure 11. Figure 11a shows that numerous dimples were observed on the PAGBs, exhibiting quasi-cleavage fracture characteristics at an interlayer temperature of 100 °C. In contrast, dimples were not observed on the fracture surface at 200 °C, and the fracture surface exhibited cleavage fracture features, as shown in Figure 11b. Figure 12 shows the phase maps of the components under different interlayer temperatures. A higher proportion of residual austenite was formed at an interlayer temperature of 100 °C, as shown in Figure 12a. A faster cooling rate hinders the uniform diffusion of carbon within the austenite and enhances the stability of the austenite. Garrison et al. [53] have shown that dimple ductile fracture is associated with void nucleation, growth, and coalescence. The mechanical induction of martensitic transformation in stable retained austenite occurs [54]. This is due to the transformation-induced plasticity (TRIP) effect of retained austenite. During plastic deformation, significant stress and dislocation concentrations arise at the interfaces between tempered martensite and newly formed hard martensite, where micro-void nucleation primarily takes place [55,56]. Furthermore, the presence of film-like retained austenite can effectively alleviate stress concentrations at crack tips, improving the low-temperature toughness of the component.

## 4. Conclusions

In this study, HSLA thin-walled components were additively manufactured by SAAM. The microstructure and mechanical properties of the components manufactured at different interlayer temperatures were characterized based on the same heat input. The conclusions are detailed below.

The microstructures of the fabricated components are distinctly different as the interlayer temperature increased, for instance, BF, constituting the primary phase at low interlayer temperatures, and PF, dominating at high interlayer temperatures. This shift is primarily attributed to the decrement in cooling rate as interlayer temperature increases, which results in a transition from non-diffusional phase transformation to diffusional phase transformation in the microstructure.

The fabricated components exhibit almost no orientation-dependent microstructure, mainly because the microstructure undergoes multiple allotropic transformations.

The hardness, strength, and toughness of components gradually decrease as the interlayer temperature rises. Hardness and strength exhibit a significant strong correlation, while toughness tends to scatter more under the influence of the critical region.

The cooling rate can effectively control the type of solid-state phase transformation. A cooling rate lower than 4.22 °C/s tends to form large polygonal ferrite, while a cooling rate near 6.12 °C/s favors the formation of fine bainitic ferrite. However, excessively high cooling rates, greater than 10 °C/s, are prone to forming brittle phases such as M-A constituents.

## Figures and Tables

**Table 1:** Chemical composition of the GWR-WEF1 wire electrode (wt%).

C	Si	Mn	P	S	Cr	Mo	Ni	Cu	Fe
0.115	0.22	1.44	0.010	0.005	0.03	0.46	1.16	0.03	Bal.

**Table 2:** Optimized parameters used in SAAM.

Current (A)	Voltage (V)	Scanning Speed (mm/min)	Contact Tip–Substrate Distance (mm)	Heat Input (J/mm)	Interlayer Temperature (°C)
575	31.5	750	20	1449	100
575	31.5	750	20	1449	200

**Table 3:** Mechanical parameters at different interlayer temperatures.

Interlayer Temperature (°C)	YS (MPa)	UTS (MPa)	EL (%)
100	564 ± 3	758	32.8
200	504 ± 15	740	21.6

## Data Availability

The original contributions presented in the study are included in the article, further inquiries can be directed to the corresponding author.
